# Loss of expression of the double strand break repair protein ATM is associated with worse prognosis in colorectal cancer and loss of Ku70 expression is associated with CIN

**DOI:** 10.18632/oncotarget.694

**Published:** 2012-10-28

**Authors:** Andrew D Beggs, Enric Domingo, Megan McGregor, Mikael Presz, Elaine Johnstone, Rachel Midgley, David Kerr, Dahmane Oukrif, Marco Novelli, Muti Abulafi, Shirley V Hodgson, Wakkas Fadhil, Mohammad Ilyas, Ian PM Tomlinson

**Affiliations:** ^1^ Molecular & Population Genetics Laboratory and 8NIHR Comprehensive Biomedical Research Centre, Wellcome Trust Centre for Human Genetics, University of Oxford, Oxford, UK; ^2^ Department of Oncology, University of Oxford, Level 4, Academic Block, John Radcliffe Hospital, Oxford, United Kingdom; ^3^ Nuffield Department of Clinical Laboratory Sciences, University of Oxford, Level 4, Academic Block, John Radcliffe Hospital, Oxford, United Kingdom; ^4^ Department of Histopathology, Rockefeller Building, University College London, London, UK; ^5^ Division of Pathology, School of Molecular Medical Sciences, University of Nottingham, Queen's Medical Centre, Nottingham, UK; ^6^ Department of Surgery, Croydon University Hospital, Croydon, UK; ^7^ Department of Medical Genetics, St George's University of London, Cranmer Terrace, Tooting, London, UK

**Keywords:** Double strand break repair, ATM, γ-H2AX, colorectal cancer, prognosis

## Abstract

Repair of double strand DNA breaks (DSBs) is pivotal in maintaining normal cell division and disruption of this system has been shown to be a key factor in carcinogenesis. Loss of expression of the DSB repair proteins have previously been shown to be associated with poorer survival in colorectal cancer. We wished to ascertain the relationship of altered expression of the DSB repair proteins γ-H2AX (gamma-H2AX), ATM and Ku70 with biological and clinico-pathological features of colorectal cancer. 908 tumours from the VICTOR clinical trial of stage II/III colorectal cancer were analysed for expression of γ-H2AX, ATM and Ku70 using immunohistochemistry. Expression levels were correlated with CIN and with disease-free survival, correcting for microsatellite instability, BRAF/KRAS mutation status, Dukes stage, chemo/radiotherapy, age, gender and tumour location. Down-regulated Ku70 expression was associated with chromosomal instability (p=0.029) in colorectal cancer. Reduced ATM expression was an independent marker of poor disease-free survival (HR=1.67, 95% CI 1.11-2.50, p=0.015). For Ku70, further studies are required to investigate the potential relationship of non-homologous end joining with chromosomal instability. Loss of ATM expression might serve as a biomarker of poor prognosis in colorectal cancer.

## INTRODUCTION

Repair of DNA double-strand breaks (DSBs) by non-homologous end joining (NHEJ) is essential in maintaining DNA integrity [1]. The proteins that play a pivotal role in this process are ataxia telangiectasia-mutated (ATM), the histone γ-H2AX and the Ku70/Ku80 complex. Defective DSB repair may contribute to the chromosome instability (CIN) phenotype in cancer [1]. Although poorly defined, CIN is generally used as shorthand for cancers with polyploidy, grossly abnormal chromosome number and multiple structural abnormalities.

H2AX is a member of the histone H2A family that helps to package and organise DNA into chromatin, the basic subunit of which is the nucleosome [2]. ATM is a serine-threonine kinase that is activated by DSBs and activates several downstream targets, including those involved in DNA repair. It is also, however, involved in induction of cell senescence and apoptosis [3]. ATM and the related proteins ataxia telangiectasia Rad-3 related (ATR) and DNA-dependent protein kinase (DNA-PK) are activated immediately when a double-strand DNA break occurs. These then act to phosphorylate H2AX at its fourth serine residue, a species that is termed γ-H2AX (gamma-H2AX) [2]. ATM activation is mediated by autophosphorylation of the serine residue at position 1981 in the ATM protein [4]. This is in turn mediated by mobilisation of the Rad50/MRE complex.g-H2AX then binds near the site of the DSB and enters a signal amplification loop. NBS1 (Nijmegen breakage syndrome protein 1) and MDC1 (Mediator of DNA damage checkpoint protein 1) – both directly involved in DSB repair – are recruited, stimulate ATM expression and further increase H2AX phosphorylation and recruitment [5]. The bound NBS1 then goes on to activate DSB repair, which can occur via either homologous recombination (HR) or non-homologous end joining (NHEJ). γ-H2AX also has several roles in regulating apoptosis, presumably if DSB repair fails [6]. Ku70 and Ku80 form the Ku heterodimer complex that binds to the site of DSB and aids in NHEJ.

Few studies have examined the expression of DSB repair proteins in colorectal carcinogenesis, in particular their relationships with chromosomal instability (CIN) or association with patient outcome. Grabsch et al [7] analysed 342 colorectal cancers for expression of ATM, BRCA1, BRCA2, Ku70 and Ku80. They found that patients whose tumours had normal expression of BRCA1 or ATM had significantly longer survival, with ATM being an independent prognostic marker. Rigas et al [8] found reduced expression of Ku70/80 in colorectal adenomas. Komuro et al [9] found that relatively high Ku70/80 expression was associated with longer disease-free survival and increased radiosensitivity in a set of 96 rectal cancers. No previous studies have examined the role of γ-H2AX in colorectal cancer in this way, and none that have compared DSB repair protein expression with the presence of CIN.

In this study, we examined the expression of g-H2AX, ATM and Ku70 in a set of 908 stage II/III colorectal cancers. We searched for associations between DSB repair protein expression and chromosomal instability (CIN). Finally, we tested each protein as a marker of prognosis.

## RESULTS

### γ-*H2AX*, *ATM* and *Ku70* expression

A total of 2,821 tissue cores from 908 cancers on 24 tissue microarrays were available from the VICTOR study. Owing to tissue loss during the IHC process and limited availability of some blocks, all cores were stained for g-H2AX, 2464 for ATM and 1195 for Ku70. Because of this, not every case had three evaluable cores. Image cytometry was performed on 469 of the 908 cancers: for γ-H2AX, 385 tumours with both IHC and ploidy analysis were successfully analysed; for ATM, 394 tumours were studied; and for Ku70, 204 tumours were analysed.

In order to determine the baseline level of γ-H2AX expression and therefore, by inference, double strand breaks (DSB), nuclear expression of γ-H2AX was initially assessed in 411 normal tissue cores from different patients. None of the samples showed nuclear g-H2AX expression. Of the 908 carcinomas, 440/908 (48.5%) had nuclear γ-H2AX expression. For ATM, 537/908 (59.1%) cancers had reduced/absent expression compared to normal mucosa and for Ku70, 443/908 (48.8%) cancers had reduced/absent expression. There was no association between the expression levels of H2AX and ATM ($\chi_{1}^{2}=0.43$, p=0.51), H2AX and Ku70 ($\chi_{1}^{2}=0.0013$, p=0.971) or ATM and Ku70 ($\chi_{1}^{2}=1.65$, p=0.20). Inter-observer agreement was high in all cases (for γ-H2AX, Spearman's γ=0.71, p<0.001; for ATM, γ=0.80, p<0.001); and for Ku70,γ= 0.89, p<0.001).

### DSB repair protein expression, CIN and clinicopathological variables

We tested for associations between DSB repair protein expression and CIN (Table 1). Evidence of an association was found between loss of expression of Ku70 and CIN ($\chi_{1}^{2}=4.77$, p=0.029), but none was found for γ-H2AX ($\chi_{1}^{2}=0.23$, p=0.63) or ATM ($\chi_{1}^{2}=0.013$, p=0.91). We also tested expression of each protein against all other clinicopathological variables (age, gender, Dukes stage, post-operative chemotherapy, pre-operative radiotherapy, site of tumour, *KRAS* mutation status, *BRAF* mutation status, MSI and trial group, i.e. placebo/rofecoxib). There was no significant association after correcting for multiple testing (p>4.53×10^−3^ in all cases), and there was specifically no association between DSB protein expression and rofecoxib therapy during the trial.

**Table 1:** Comparison of DSB seen vs presence of chromosomal instability demonstrated by ploidy status, demonstrating no relationship between number of double strand breaks seen and chromosomal instability status

	Double stranded breaks			
γ-H2AX				p
	Absent	Present	Total	0.66
CIN+	143	115	258	
CIN-	100	87	187	
Total	243	202	445	
**ATM**				
	**Absent**	**Present**	**Total**	**0.91**
CIN+	52	107	159	
CIN-	103	207	310	
Total	158	314	469	
**Ku70**				
	**Absent**	**Present**	**Total**	**0.029**
CIN+	20	65	85	
CIN-	54	90	144	
Total	74	155	229	

### DSB protein expression and prognosis

In total, 2,434 people were recruited to the VICTOR trial. Patients were randomly assigned in a double blind fashion to the trial at a variable time (mean 193 days, range 4-386 days) after completing definitive treatment (surgery and/or chemo/radiotherapy). 1,217 individuals were randomised to the rofecoxib arm (one 25mg tablet of rofecoxib daily) of the trial and 1,217 to the placebo arm. Of the 908 patients available for this study, the average overall survival (OS) was 4.81 years (range 0.3-7.86 years) and the average disease-free survival (DFS) was 3.36 years (range 0.3-7.86 years).

**Figure 1:**
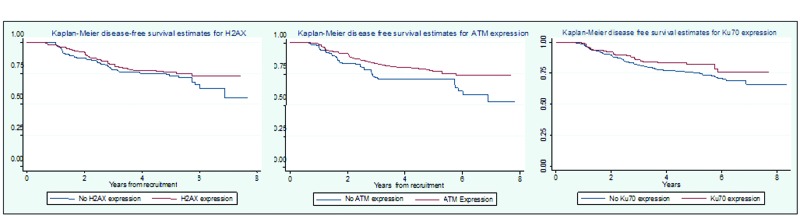
Kaplan-Meier plots showing relationship between disease free survival and g-H2AX, ATM and Ku70 expression

Kaplan-Meier plots of disease-free survival (DFS) by g-H2AX, ATM and Ku70 status are shown in Figure 1. Univariate Cox regression modelling showed that worse survival was associated with reduced expression of ATM in tumours as compared to normal tissue (HR=1.56, 95% CI 1.05-2.33, p=0.028). No significant associations were found between survival and expression of g-H2AX (HR=1.23, 95% CI 0.83-1.82, p=0.30) and Ku70 (HR=1.41, 95% CI 0.91-2.13, p=0.12). We also found no association between loss of Ku70 expression and disease free survival within the CIN tumour group (HR 1.42, 95% CI 0.59-3.44, p=0.433) in a univariate model.

We then performed multivariate reverse stepwise Cox regression modelling (with a threshold for removal from the model of p>0.05) for each of g-H2AX, ATM and Ku70, including as co-variates age, gender, Dukes stage, tumour location, trial arm (rofecoxib vs. placebo), chemotherapy status, radiotherapy status, CIN, *KRAS* and *BRAF* mutation status and MSI (Table 2). Reduced expression of ATM remained associated with DFS (HR=1.67, 95% CI 1.11-2.50, p=0.015), as was Dukes stage C (HR=2.53, 95% CI 1.55-4.11, p<0.001). Neither g-H2AX nor Ku70 expression was associated with DFS in the multivariate model (details not shown). In univariate analysis we found that chemotherapy (HR 0.50, 95% CI 0.35-0.72, p<0.001) was associated with better DFS, but not radiotherapy (HR 0.68, 95% CI 0.44-1.05, p=0.086) probably due to the small numbers of patients undergoing radiotherapy.

**Table 2:** Table of clinicopathological variables and their correlations with gamma-H2AX, ATM and Ku70 expression corrected for multiple testing via Bonferroni method

		γ-H2AX			ATM			Ku70		
		Not expressed	Expressed	P	Not expressed	Expressed	P	Not expressed	Expressed	P
Study arm	Placebo	91	89	0.260	71	123	0.084	34	72	0.244
	Rofecoxib	116	89		56	144		40	58	
Chemo	No	69	72	0.148	40	96	0.384	24	52	0.282
	Yes	138	106		87	171		50	78	
RXT	No	181	161	0.350	115	236	0.520	68	116	0.539
	Yes	26	17		12	31		6	14	
Dukes stage	B	84	91	0.038	54	112	0.914	35	70	0.368
	C	123	87		73	115		39	60	
Gender	Female	67	67	0.279	45	94	0.965	29	54	0.743
	Male	140	111		82	173		45	76	
Site	Colon	139	112	0.385	85	175	0.786	46	85	0.644
	Rectum	68	66		42	92		28	45	
KRAS status	Wild type	125	112	0.590	87	162	0.127	47	77	0.482
	Mutant	68	54		33	89		23	47	
BRAF status	Wild type	166	147	0.472	108	217	0.332	63	104	0.236
	Mutant	27	19		12	34		7	20	
MSI status	MSS	167	153	0.087	109	224	0.637	66	107	0.085
	MSI	26	13		11	27		4	17	
Age (95% CI)	-	64.6yrs (63.4-65.9)	63.2yrs (61.8-64.6)	0.145	65.2yrs (63.6-66.7)	63.5yrs (62.3-64.6)	0.097	62.6yrs (60.4-64.8)	63.5yrs (61.8-65.2)	0.517

In order to explore whether there was a single model that could be derived based on a combination of the expression levels of all DSB repair proteins, a multivariate reverse stepwise Cox regression model was constructed with the following variables: γ-H2AX, ATM, Ku70 and the above co-variates. Loss of ATM expression was independently associated with DFS (HR=1.92, 95% CI 1.03-3.57, p=0.003) as well as Dukes C stage (HR 2.34, 95% CI 1.43-3.83, p=0.001). For comparison, the same analysis was carried out for overall survival, but no variable was a significant predictor of outcome, perhaps owing to fewer events.

Finally, in order to explore whether DSB repair protein expression was related to response to radiotherapy and chemotherapy as measured by DFS, all patients undergoing radiotherapy (n=68) and chemotherapy (n=436) were analysed via a univariate Cox regression model. In the radiotherapy group, there was a tendency towards longer DFS in patients with loss of expression of g-H2AX however there was no significant relationship between disease free survival and loss of expression of γ-H2AX (HR=2.92, 95% CI 0.94-9.14, p=0.064), ATM (HR=1.78, 95% CI 0.63-5.03, p=0.28) or Ku70 (HR=0.58, 95% CI 0.07-4.98,p=0.62). In the chemotherapy group, there was no significant associations between disease free survival and loss of expression of γ-H2AX (HR 1.28, 95% CI 0.82-2.00, p=0.277), ATM (HR 1.52, 95% CI 0.95-2.43, p=0.078) or Ku70 (HR 1.03, 95% CI 0.54-1.96, p=0.936), although loss of expression of ATM came close to significance.

**Table 3:** Table demonstrating relationship between disease free survival and all variables in a multivariate survival model, before stepwise correction

	Disease free survival		
Variable	HR	95% CI	P-value
g-H2AX expression (1=no expression)	0.84	0.42-1.68	0.630
ATM expression (1=no expression)	0.54	0.27-1.09	0.087
Ku70 expression (1=no expression)	0.78	0.40-1.54	0.477
Gender (1=male)	1.06	0.49-2.30	0.874
Trial arm (1=Rofecoxib)	0.88	0.43-1.80	0.731
Stage (1=Dukes C)	0.70	0.28-1.72	0.436
Age (years)	0.99	0.96-1.03	0.702
Time from diagnosis to recruitment (days)	1.00	0.99-1.01	0.187
Chemotherapy (1=yes)	0.99	0.09-10.53	0.990
Radiotherapy (1=yes)	0.99	0.31-3.18	0.996
Site (1=rectum)	0.75	0.33-1.72	0.502
CIN status (1=CIN+)	1.32	0.65-2.71	0.440
KRAS mutation(1=mutant)	0.78	0.37-1.63	0.509
BRAF mutation (1=mutant)	1.06	0.67-3.09	0.907
MSI status (1=MSI+)	0.55	0.11-2.63	0.450

## DISCUSSION

We have carried out a large-scale investigation of the expression of the double strand break repair proteins γ-H2AX, ATM and Ku70 in colorectal cancer. We found that chromosomal instability was associated with loss of expression of Ku70, but that there was no association with either γ-H2AX or ATM. It is unclear as to why all DSB proteins do not have altered expression in CIN, although we note that in a mouse model, hepatocellular carcinomas from *Ku70* −/− knockout mice displayed greater CIN [13] than those from their wild type littermates.

We have also confirmed the findings of Grabsch et al [7] who found that reduced ATM expression was associated with worse survival in colorectal cancer patients receiving adjuvant therapy. We found that the association of disease-free survival with ATM expression is independent of tumour stage, location, use of chemo/radiotherapy, gender, *KRAS/BRAF* mutation status, microsatellite instability or chromosomal instability. Although we found associations between chemotherapy/radiotherapy use and disease free survival on univariate analysis, this was not seen on multivariate analysis, probably due to interactions with our genetic markers.

The association between CIN and poorer survival in colorectal cancer is well documented [14]. Komuro et al [9, 15] found an association between higher Ku70 expression and poorer prognosis in rectal and advanced colorectal cancers. Although we did not find a statistically significant association between Ku70 expression and poorer DFS in our stage II/III cancer cases, there was a tendency towards reduced DFS in patients with loss of Ku70 expression. Our findings disagree with those of Komouro and our study has a larger number of cancers and was not restricted to rectal cancers only. It is possible that we did not detect a significant association due to the relatively small numbers of samples successfully analysed for Ku70 compared with the other markers.

The lack of association in our study between ATM or γ-H2AX expression and CIN is of interest. Celeste el al [16] demonstrated that H2AX −/− mice demonstrated chromosomal instability in embryonic fibroblast cultures in a similar pattern to ATM −/− and Ku70/80 −/− mice. Van Gent et al [17] demonstrated that CIN can occur either because of faulty cell cycle checkpoint regulation or failure of DNA repair. ATM and γ-H2AX signal tend to act through HR, whereas Ku70 acts via NHEJ. It is possible that the type of chromosomal instability seen in colorectal cancer is principally associated with NHEJ.

The induction of DSBs is one mechanism [18] by which radiotherapy induces cell death. We have shown that loss of γ-H2AX expression has a tendency towards worse survival in colorectal cancer in those patients who have undergone pre-operative radiotherapy, thus suggesting that DSB repair deficient tumours may be non-responders to radiotherapy. This conclusion is based on a small sample size, and must be regarded as tentative until a larger scale study can be set up to study this phenomenon. A similar phenomenon has been seem in patients undergoing chemotherapy with loss of ATM expression within their tumours, showing a tendency towards worse survival in colorectal cancer.

In conclusion, we have demonstrated an association between presence of Ku70 and CIN in colorectal cancer and confirmed an association between ATM expression and superior DFS. These associations are independent of other variables. For Ku70, functional analyses will determine whether the association is related to the underlying cause of CIN lying in increased DSBs and hence NHEJ activation. By comparison, ATM expression might be used in the near future as a biomarker of prognosis in colorectal cancer.

## METHODS

### Patients and tissue microarray (TMA)

Patients were recruited from the Vioxx in Colorectal Cancer study (VICTOR) study [10]. VICTOR was a randomised control trial comparing the effect of post-treatment rofecoxib against placebo in reducing recurrence in patients who had undergone a resection of a potentially curable stage II or stage III colorectal carcinoma. The VICTOR trial initially aimed to recruit 7,000 patients, but it was terminated prematurely in 2004 due to the adverse cardiovascular risk and mortality associated with COX-2 inhibitors. In total 2,434 patients were recruited and, of those, 978 had tumour blocks and/or blood samples available for study. Patients were randomised into two groups, one group taking 25mg rofecoxib daily and the other placebo. The median duration of rofecoxib therapy was 7.4 months. Twenty-four tissue microarrays were constructed from tumour and normal tissue from each patient. At least three representative 0.6mm cores of tumour and one paired 0.6mm core of normal tissue (obtained from full thickness normal colon 10cm distal to the tumour) were sampled from each patient, as well as at least two cores of metastasis and/or lymph node if present. For immunohistochemistry, serial 4μM sections were cut from each block.

### Immunohistochemistry

For gamma-H2AX, a mouse monoclonal antibody was used (Abcam ab22551) at 1:50 dilution and 1 hour incubation at room temperature, for ATM a rabbit monoclonal antibody was used (Abcam ab32420) at 1:50 dilution with overnight incubation at 4°C, and for Ku70 a mouse monoclonal antibody (Abcam ab2172) at 1:50 dilution with overnight incubation at 4°C. A standard indirect immunohistochemistry (IHC) technique was used for all three antibodies. The tissue section was dewaxed in xylene and rehydrated in graded alcohol solutions. Endogenous peroxidase activity was blocked by immersing the slides in 1% H_2_O_2_ in methanol for 15 minutes. Heat-induced epitope retrieval was then carried out in a pressure cooker using 1L of citrate buffer solution (pH 6) boiled at full pressure for 3 minutes. Slides were then incubated in horse serum (Vectalabs) for 30 minutes as a blocking step and washed with PBS. Next, the slides were incubated with the primary antibody, washed with PBS and a secondary biotinylated horse anti-mouse antibody (Vectalabs) was applied at room temperature for 1 hour. Slides were then washed with PBS, incubated with an avidin-biotin-horseradish peroxidase solution (VectaStain ABC Elite) for 1 hour and then washed again with PBS. Slides were then stained using DAB solution (Sigma Fast-DAB) for 2 minutes (experimentally determined), washed with PBS and counterstained with haemotoxylin. Sections were finally dehydrated in serial alcohol solutions and coverslips were applied. Positive controls consisted of human breast carcinoma (ATM), human tonsil tissue (Ku70) and human tonsillar lymphoma (g-H2AX) as recommended by the antibody manufacturer's datasheet. Negative controls consisted of tissue through which all steps were performed except application of the primary antibody.

A Carl-Zeiss NanoZoomer scanning microscope was used to create a digital image of each slide at 40x optical magnification. Images were transferred to a Genetix SlidePath TMA management system. Expression scoring was carried out by two independent observers (MP and MMcG) and compared to the expression seen in the positive control. As there were three cores for each tumour, each core was scored separately and the average expression between cores for each tumour used. For g-H2AX, expression was dichotomised into either no nuclear expression (absent) or nuclear expression (>1+ nucleus per core). For ATM and Ku70/80, expression was graded as either reduced/absent (compared to normal mucosa) or present. If there was disagreement between observers for the average score across all the cores, the higher expression score was used as the assumption in this study was that loss of expression was more significant and so using the sample with least expression would skew the results. Correlation of scores between the independent observers was calculated using Spearman's Rank correlation coefficient. Representative images for IHC are shown in the supplementary materials as figures S1-S3 (available on-line)

### Tumor genotyping

Image cytometry to analyse CIN was carried out in a sub-set of tumours as described by Leedham et al [11]. We defined CIN as a modal DNA content that was distinct from and greater than the diploid peak in non-tumour cells. Ploidy was called based on the ABCDE technique of Buhmeida et al [12]. DNA for tumour genotyping was obtained from 4μM formalin fixed, paraffin embedded sections from the same block from which the TMA core was originally taken. Needle dissection was carried out under light microscopy to minimise contaminating normal tissue. Dissected tissue then underwent Proteinase K digestion, purification using Qiagen DNEasy clean-up kit and underwent quality control using a NanoDrop spectrophotometer, Microsatellite instability (MSI) status was ascertained using BAT25/BAT26 primers in a standard PCR reaction. MSI was said to be present if additional alleles were present compared to normal controls. *KRAS* and *BRAF* mutations were identified by direct sequencing (primer sequences and conditions available on request).

### Statistical analysis

To compare gamma-H2AX, ATM and Ku70 expression with ploidy, Fisher's exact and *X*^2^ tests were used. To compare survival time data for patients, Kaplan-Meier plots for survival were constructed, and for overall survival modelling, univariate and multivariate Cox proportional hazards survival models were constructed. The validity of the proportional hazards assumptions were confirmed using a proportional hazards plot and by testing Schoenfeld residuals (details available on request).

Available variables for multivariate analysis were age, gender, Dukes stage, tumour location (colon or rectum), chemotherapy, radiotherapy, CIN status, *KRAS* mutation, *BRAF* mutation and MSI.

## FUNDING

ADB was funded by a Mason Medical Foundation Research Fellowship and grants from the Peel Medical Research Trust and St Georges Hospital Charity; IPMT acknowledges funding from Cancer Research UK. The Wellcome Trust Centre for Human Genetics receives core support from the Wellcome Trust (090532/Z/09/Z).

## Supplementary Figures


