# Supplementary material for: Loss of expression of the double strand break repair protein ATM is associated with worse prognosis in colorectal cancer and loss of Ku70 expression is associated with CIN
Source: Oncotarget. 2012 Oct 28;3(11):1348–55. doi: 10.18632/oncotarget.694 (PMC3717797; doi:10.18632/oncotarget.694)

Loss of expression of ATM is associated with worse prognosis in colorectal cancer and loss of Ku70 expression is associated with CIN – Beggs et al

Figure S1: H2AX representative sections for IHC scoring

Positive:

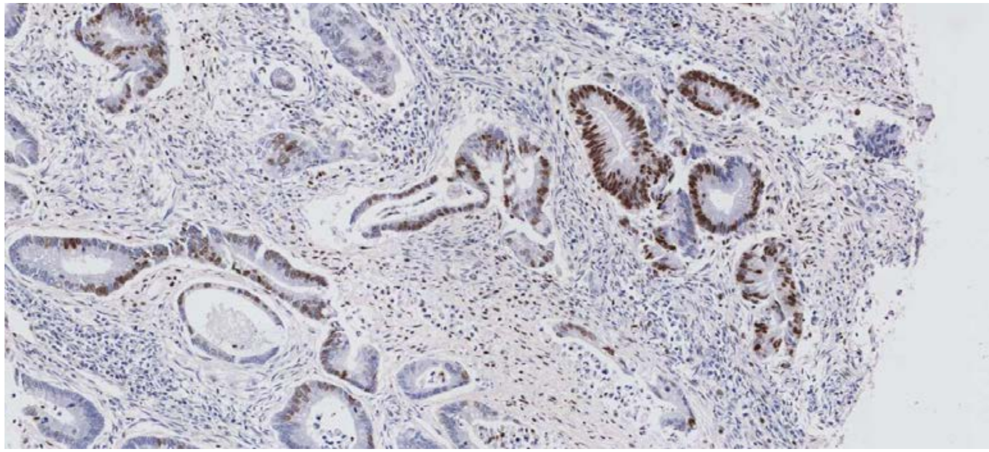

Negative:

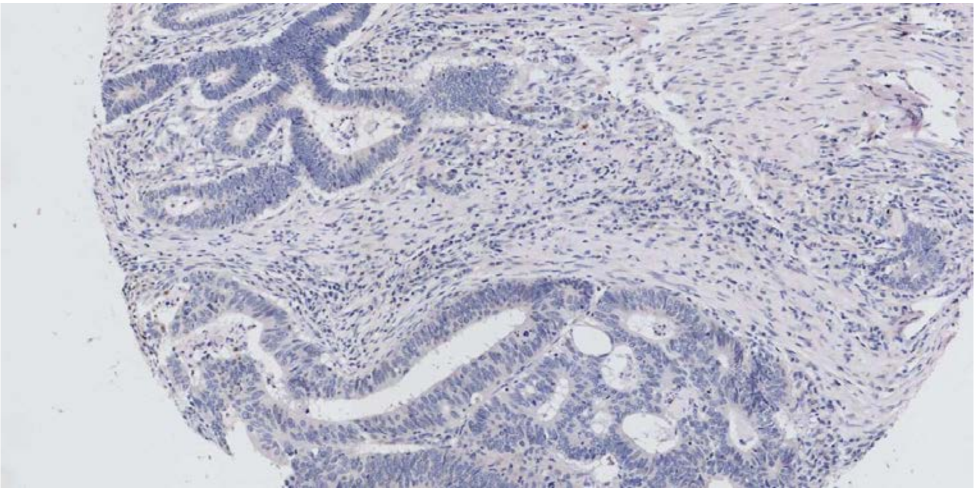

Figure S2: ATM representative sections for IHC scoring

Positive (left image = low power, right image = high power):

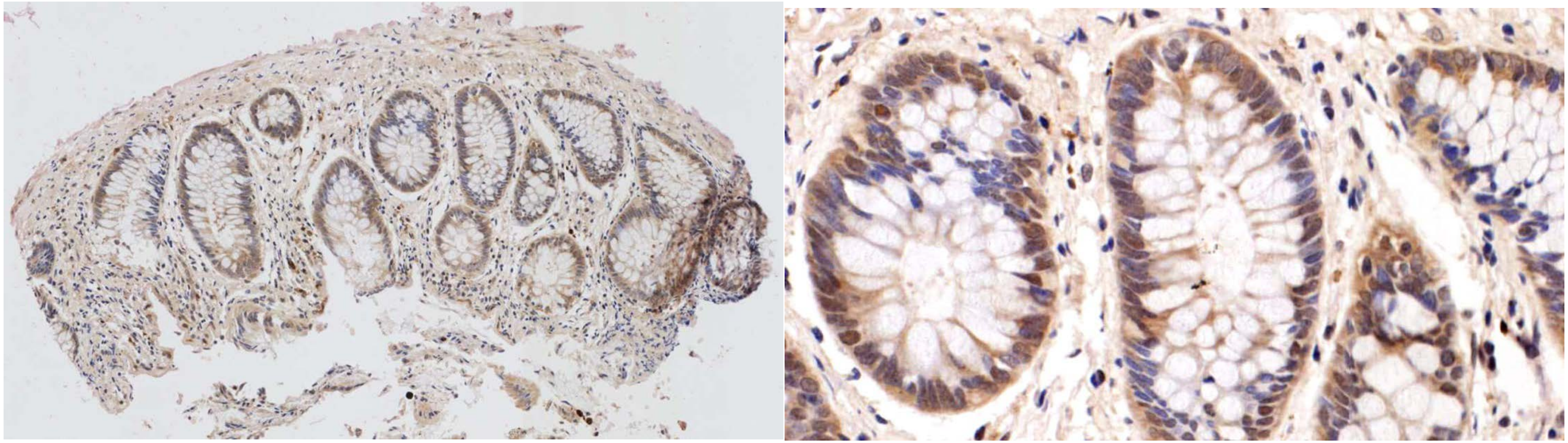

Negative (left image = low power, right image = high power)

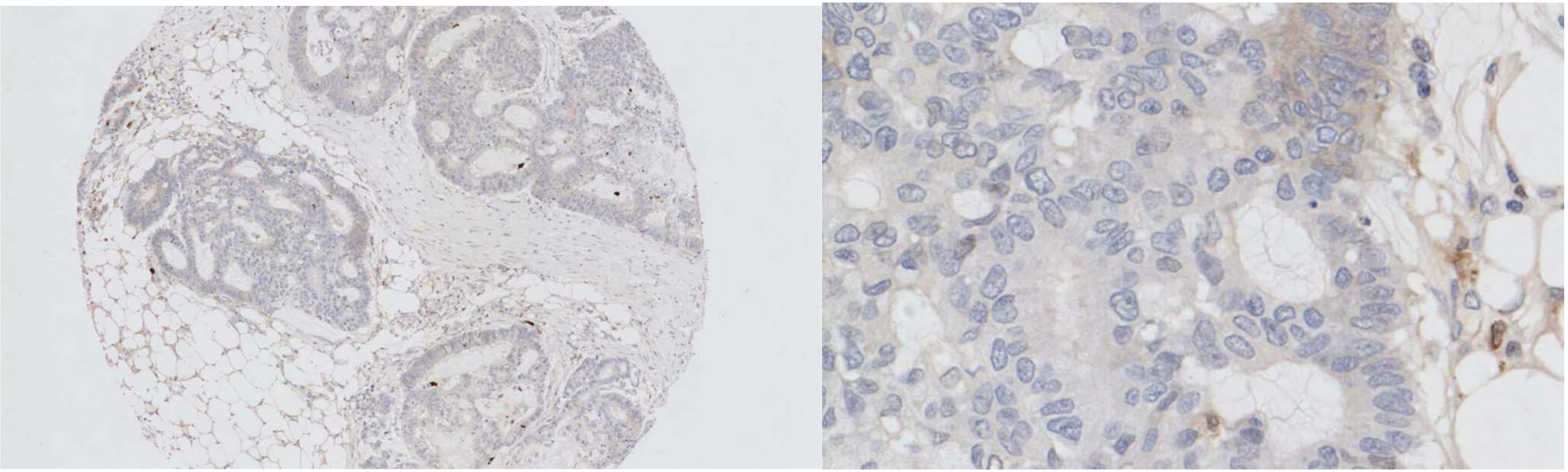

Figure 3: Ku70 representation sections for IHC scoring

Positive:

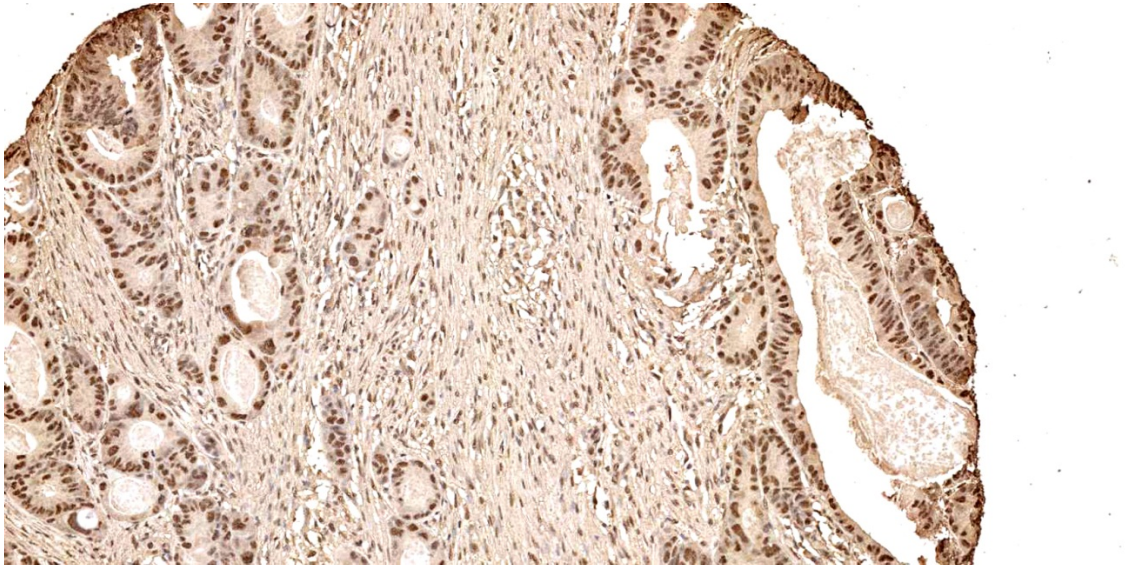

Negative:

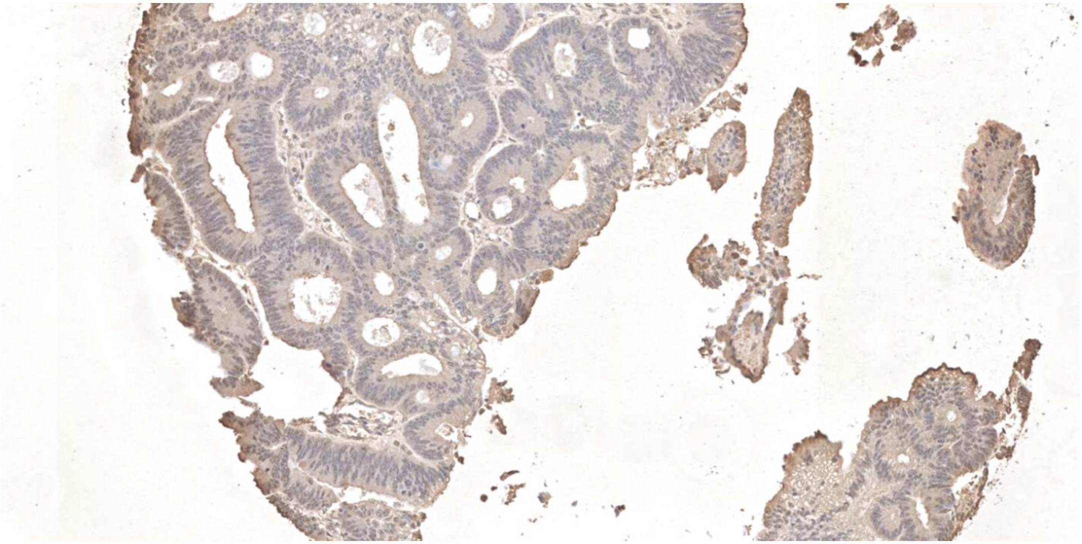

Supplement: Supplementary file 1 [file oncotarget-03-1348-s001.pdf]
